# Increasing Lateralized Motor Activity in Younger and Older Adults using Real-time fMRI during Executed Movements

**DOI:** 10.1016/j.neuroscience.2017.02.010

**Published:** 2018-05-15

**Authors:** Heather F. Neyedli, Cassandra Sampaio-Baptista, Matthew A. Kirkman, David Havard, Michael Lührs, Katie Ramsden, David D. Flitney, Stuart Clare, Rainer Goebel, Heidi Johansen-Berg

**Affiliations:** a Centre for Functional MRI of the Brain, Nuffield Department of Clinical Neurosciences, University of Oxford, Oxford, UK; b School of Health and Human Performance, Dalhousie University, Halifax, Nova Scotia, Canada; c Department of Cognitive Neuroscience, Maastricht University, The Netherlands Brain Innovation B.V., Maastricht, The Netherlands

**Keywords:** BOLD, Blood-oxygen-level-dependent, FWHM, full width at half maximum, GLM, general linear model, LI, laterality index, NF, Neurofeedback, PSC, percent signal change, ROI, region of interest, TR, repetition time, tDCS, transcranial direct current stimulation, TCP, transmission control protocol, neurofeedback, motor cortex, ageing, stroke, real-time fMRI

## Abstract

Neurofeedback training involves presenting an individual with a representation of their brain activity and instructing them to alter the activity using the feedback. One potential application of neurofeedback is for patients to alter neural activity to improve function. For example, there is evidence that greater laterality of movement-related activity is associated with better motor outcomes after stroke; so using neurofeedback to increase laterality may provide a novel route for improving outcomes. However, we must demonstrate that individuals can control relevant neurofeedback signals. Here, we performed two proof-of-concept studies, one in younger (median age: 26 years) and one in older healthy volunteers (median age: 67.5 years). The purpose was to determine if participants could manipulate laterality of activity between the motor cortices using real-time fMRI neurofeedback while performing simple hand movements. The younger cohort trained using their left and right hand, the older group trained using their left hand only. In both studies participants in a neurofeedback group were able to achieve more lateralized activity than those in a sham group (younger adults: *F*(1,23) = 4.37, *p* < 0.05; older adults: *F*(1,15) = 9.08, *p* < 0.01). Moreover, the younger cohort was able to maintain the lateralized activity for right hand movements once neurofeedback was removed. The older cohort did not maintain lateralized activity upon feedback removal, with the limitation being that they did not train with their right hand. The results provide evidence that neurofeedback can be used with executed movements to promote lateralized brain activity and thus is amenable for testing as a therapeutic intervention for patients following stroke.

## Highlights

• Healthy adults performed movements while receiving neurofeedback from real-time fMRI.
• Two experiments were performed, one with younger and one with older adults.
• Neurofeedback (NF) represented the laterality of activation in the motor cortices.
• The NF groups produced more lateralized activity than the sham group.

## Introduction

There are a range of neurorehabilitation techniques that have been developed to facilitate motor recovery after stroke such as physiotherapy, motor imagery and non-invasive brain stimulation (Allman et al., 2016, Barclay-Goddard et al., 2011, French et al., 2007, Hao et al., 2013, Pollock et al., 2007, Sirtori et al., 2009, Thieme et al., 2013). Following stroke, movement of the affected limb is associated with increased activity in the unaffected motor cortex and hence bilateral activation of motor regions (Cramer et al., 1997, Gerloff et al., 2006, Nelles et al., 1999). Furthermore, worse motor function is associated with a more bilateral activation pattern (Johansen-Berg et al., 2002, Ward et al., 2003) thus rehabilitation interventions that rebalance brain activity to a more lateralized or contralateral pattern may enhance therapy outcomes (Ward and Cohen, 2004). For example, improvements in motor function with transcranial direct current stimulation (tDCS) are accompanied by increased activity in contralateral (ipsilesional) sensorimotor cortex (Stagg et al., 2012, Allman et al., 2016). An alternative intervention that may promote the lateralization of brain activity and thus lead to beneficial plasticity following stroke is neurofeedback. Neurofeedback involves measuring an individual’s brain activity and displaying it back to them in an intuitive format. The individual can then be asked to use this feedback display to alter their brain activity with the aim of inducing plasticity and improved function. Thus, there is potential for stroke patients to use neurofeedback training to lateralize brain activity following stroke.

Neurofeedback can be used in rehabilitation to enhance motor imagery which may promote more lateralized activity (e.g., Auer et al., 2015, Chiew et al., 2012, deCharms et al., 2004, Hampson et al., 2011, Yoo et al., 2008). For example, previous research using motor imagery has shown that healthy participants can use neurofeedback from real-time fMRI (rtfMRI) to increase activation in the hemisphere contralateral to imagined movement (deCharms et al., 2004, Yoo et al., 2008). However, there is variability in efficacy: Chiew et al. (2012) showed that only a sub-set of their healthy participants (6 of 13 participants) could modulate the laterality of activity between hemispheres using motor imagery. With a longer training period of twelve sessions, Auer et al. (2015) demonstrated that 25 of 32 participants could control a laterality index using motor imagery. Although motor imagery provides a useful paradigm for neurofeedback, some people struggle to carry out motor imagery tasks and it is difficult to assess neurofeedback performance which may be confounded with motor imagery ability.

Relatively few studies to date have analyzed the benefits of neurofeedback in conjunction with executed movement to promote lateralization. This is important because, although more than 75% of stroke patients have motor disability following stroke (Lawrence et al., 2001), many retain at least some movement in the affected limbs and much of the movement rehabilitation received after stroke focuses on executed movements. This group could benefit from neurofeedback combined with physical practice, which may be more effective than neurofeedback with motor imagery. The association between real hand movements and increased spread of cortical activity with neurofeedback has been demonstrated elsewhere (Yoo and Jolesz, 2002) but in this study no comparison was made to a no feedback or sham condition. Overall, more evidence is needed on capacity to modulate neurofeedback signals during executed movement.

When considering the application of neurofeedback to patients with stroke, it is important to note that most (c.f., Sitaram et al., 2012) prior rtfMRI neurofeedback studies were performed in healthy younger adults, however the condition largely affects older individuals (Lawrence et al., 2001). There is evidence that age affects factors such as the hemodynamic response, neuronal and glial responsiveness, and scope for plasticity (Di et al., 2014, Freitas et al., 2011, Gauthier et al., 2013, Leal and Yassa, 2013, Thomas et al., 2014), which means that older individuals may respond differently to rtfMRI tasks. Indeed, it is known that older individuals tend to perform worse on cognitive tasks than their younger counterparts, with fMRI data supporting the theory that age influences brain activity, and that brain activity is related to task performance (Steffener et al., 2014). Further, older adults also show a decline in motor performance and may show decreased motor learning of fine or complex motor skills (Voelcker-Rehage, 2008). Although there is some case evidence that older stroke patients can control imagery-related ventral pre-motor activity using neurofeedback (Sitaram et al., 2012) this is based on a study of two stroke patients and thus further supportive data are required.

The aim of the current study was to determine whether adults can increase the laterality of activation between the motor cortices while executing movements when presented with a visual representation of a laterality index (LI) measured through rtfMRI neurofeedback. Two experiments were conducted, one with healthy younger adults (age range 20–32 years) and one in healthy older adults (age range 50–77 years). In each experiment, participants were split into two groups: one which received real neurofeedback and one which received sham feedback. If participants are able to lateralize brain activity while performing physical movements, participants in the neurofeedback group should have a larger LI magnitude than participants in the sham group. Following training, a scan where no neurofeedback was present was also included to determine if participants could maintain the increased laterality in the absence of feedback.

## Experiment I methods

### Participants

Twenty-six (seven male), right-handed participants aged 20–32, median age 26, were recruited from the Oxford community. All participants provided informed consent in accordance with the Declaration of Helsinki and University of Oxford ethics committee approved the protocol (MSD-IDREC-C1-2012-151). Participants were randomly assigned to a neurofeedback (NF) group (*n* = 13, age range: 20–31, median age: 23, four male) or a Sham group (*n* = 13, age range: 20–32, median age: 29, three male) and were not aware of their group assignment. Further, all instructions to the participants were provided by an experimenter who was blinded to the group assignment. One participant in the NF group (male) was removed from analysis due to excessive head motion in the feedback scans.

### Procedure

Imaging was performed on a 7.0T Siemens Magnetom MRI system (Siemens, Erlangen, Germany). A structural image was acquired using a T1 weighted, MPRAGE sequence with 1 × 1 × 1 mm^3^ isotropic voxels (repetition time = 2200 ms; echo time = 2.2 ms; flip angle 7°, field of view, 192 × 192; matrix = 192 × 192). All fMRI scans were performed using a 16 slice (2 mm, no slice gap) axial plane, gradient echo planar image acquisition, with 2 × 2 mm^2^ in plane resolution (repetition time = 2000 ms; echo time = 25 ms; flip angle = 90° field of view, 220 × 220 mm; matrix = 110 × 110). A limited field of view was used so the data could be analyzed within a single repetition time (TR) to be fed back to the participant. The field of view, a slab of 32-mm depth, was placed angled parallel with the top of the brain and was deep enough to cover sensorimotor and pre-motor cortex.

Turbo-BrainVoyager software (Brain Innovation, Maastricht, The Netherlands) was used to preprocess (including online 3D motion correction) and analyze the fMRI data in real time using a recursive general linear model (Pollock et al., 2007). A transmission control protocol (TCP) based network interface plug in for Turbo-BrainVoyager was used to transfer the processed region of interest (ROI) time course data to a custom-made software tool, Turbo-Feedback, which performed neurofeedback signal calculation and presented the feedback signal back to the participants. The feedback was projected to the participant in the scanner using a 1042 × 768 pixel screen with a 75-Hz refresh rate.

Participants were given button boxes to hold in each hand, each with four buttons corresponding to the four fingers (excluding the thumb). The pre-feedback (pre-FB) scan (150 volumes) provided a functional localizer and consisted of eight 12-second tapping blocks, four blocks for each hand, alternating hands between blocks, interspersed with 24-second rest. The first block of tapping started after a 14-second rest period and a 22-second rest period followed the final tapping block. The participants saw the instructions ‘Right Tap’, ‘Left Tap’ and ‘Rest’ displayed in white on a black background. During the tap instructions participants were told to use each finger in sequence starting with their index finger and moving outward toward the little finger to press and release the button under their finger on the button box at a rate of approximately 1 Hz, and to repeat the sequence until they saw the rest instruction.

**Fig. 1:**
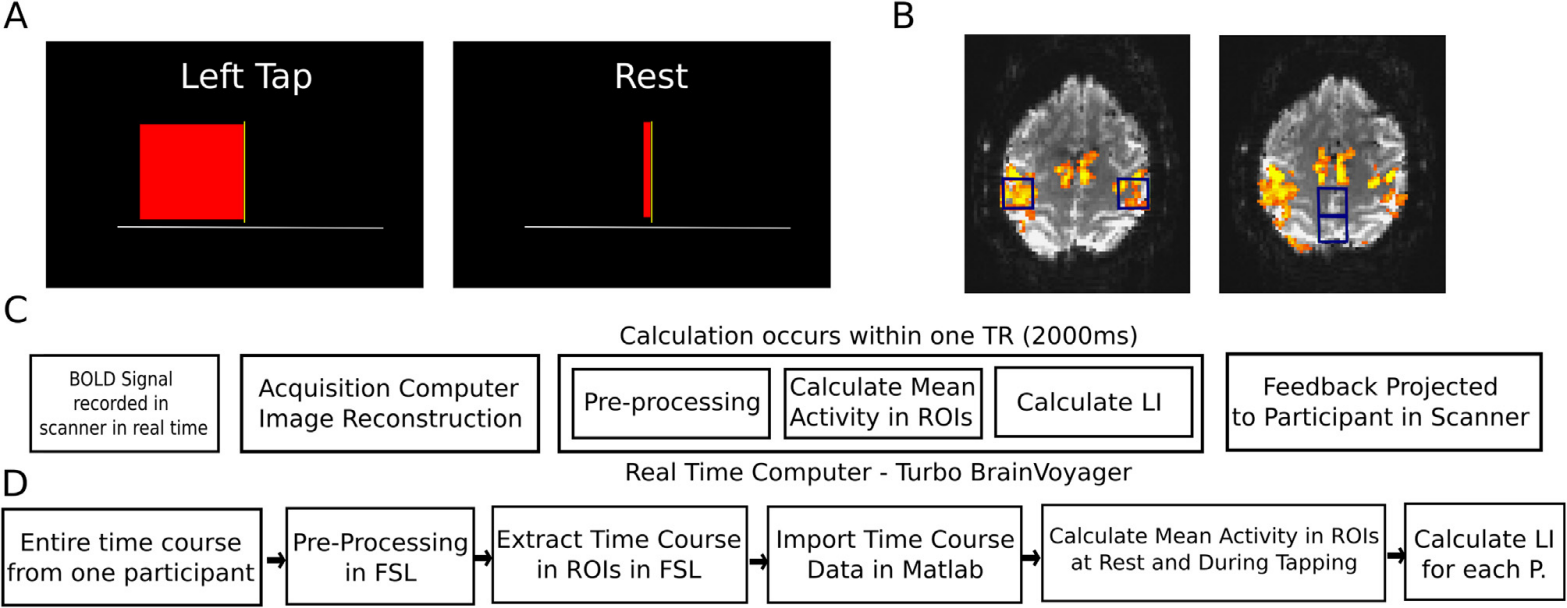
(A) Feedback display shown to participants. The bar updates in width according to the participant’s LI (see text for details). The left image presents a typical frame during the left tapping block (where the bar growing leftward represents right hemisphere lateralized activity) and the right image presents a typical frame during a rest block (where a bar close to the center represents LI close to 0 as activity is similar between the two hemispheres) (B) Image on the left shows the motor ROIs from an example participant (blue boxes) located over sensorimotor cortex. Image on the right shows sham ROIs from an example participant (blue boxes). (C) Online data processing pipeline (D) Offline data processing pipeline. Please refer to the text for details on the calculations.

The results from the real-time general linear model (GLM) analysis of the localizer scan were used to select two motor ROIs (18 × 18 × 10 mm) for each participant (regardless of group assignment), each centered over the peak of activation in the region of the hand knob in the right or left hemisphere (Fig.1B). If the participant was in the NF group, the motor ROIs would be used for neurofeedback during the experiment and subsequent data analysis. If participants were in the Sham group, the motor ROIs were only used for data analysis. For participants in the Sham group, two sham ROIs, the same size as the motor ROIs, were also selected aligned along the anterior-posterior axis centered along the midline, toward the posterior of the brain. These sham ROIs were used to provide sham feedback to the participants in the Sham group in order to provide the same visual and motivational environment as the NF group. These regions were selected to avoid voxels activated during the motor task (Fig.1B). Thus our goal was to select regions that were not involved in our task of interest (e.g., tapping). Note that the experimenter operating the real-time set up and selecting the ROIs was necessarily aware of the group assignment (NF or Sham) of the participant but the experimenter providing instructions and interacting with the participant throughout the experiment did not interact with the neurofeedback software or other components of the real-time set up and therefore was not aware of group assignment thus maintaining the double-blind study design.

Participants next took part in four FB scans during which they were instructed to perform sequential button presses with the fingers on their right or left hand, with the order of hands alternated and counterbalanced between participants. Each FB scan (180 volumes) consisted of six, 30-second blocks of finger tapping interspersed with blocks of 30-second rest. The first block of tapping started after a 20-second rest period and a 10-second rest period followed the final tapping block. Participants saw a horizontal red bar with a vertical line delineating the center point (Fig.1A). The calculation of bar width (see below) occurred within one TR and thus the width of the red bar was updated in response to Blood-oxygen-level-dependent (BOLD) signal every TR (i.e. every 2000 ms) (Fig.1A); however, for the visual feedback display the bar width smoothly increased or decreased over the course of the TR to the new value. Thus the edge of the bar appeared to smoothly and continuously move back and forth of the block over the course of the entire block rather than updating in a single jump each TR.

The equation used to calculate LI for feedback bar width during feedback was as follows:

$$\text{LI for Bar Width}=[{\text{Left ROI}}_{\text{act}}-{\text{Left ROI}}_{\text{rest}}]/{\text{Left ROI}}_{\text{rest}}-[{\text{Right ROI}}_{\text{act}}-{\text{Right ROI}}_{\text{rest}}]/{\text{Right ROI}}_{\text{rest}}$$

*where* ROI_act_ = BOLD signal in the motor ROI on the previous volume; ROI_rest_ = mean BOLD signal in the motor ROI during the previous rest block.

Note that for the Sham group, the midline sham ROIs were used in place of the Right and Left ROIs in the above equation. Thus as activity became more right hemisphere lateralized during left-handed movement, the bar would grow further to the left and vice versa for the right-handed movement. As the difference in activation between hemispheres decreased (for instance while the participant was at rest, or if there was more bilateral activation during movements), the bar shrunk toward the center.

The maximum magnitude the bar could move in either direction was initially set at one percent signal change (PSC) unit higher than the participant achieved in the most active feedback ROI during the Pre-FB scan. PSC is the difference in activation in the ROI between the tapping blocks and rest. In other words, if the PSC signal change was 1.5% in one ROI and 2% in the other ROI, the maximum bar width on each side of the bar would be set at 3%. During each feedback run, if the participant was consistently achieving close to the maximum bar width, the maximum width value would be set 1% higher on the next feedback scan. This was done to attempt to make the task challenging for the participant across all training blocks. Thus, the LI given in the equation above was scaled by the maximum bar width in order to display the feedback.

During the tapping instruction, participants were required to perform the tapping sequence as described for the functional localizer scan and to make the bar grow as far to the tapping side as possible (i.e., to grow to the right during the right-handed tapping scans and to the left during the left-handed tapping scans). Before the scan, a number of example strategies were suggested to participants (by the experimenter reading a standardized set of instructions), such as increasing the rate, force and amplitude of the movement as well as focusing more on the moving hand and focusing less on the non-moving hand. During the rest period, the participants were required to stop moving their hand, lie still and let the bar shrink toward the center.

The Post-FB scan (150 volumes) was identical to the pre-FB scan. No feedback was provided and only the instructions ‘Right Tap’, ‘Left Tap’ and ‘Rest’ were displayed. Participants were instructed to use the strategy that they found most successful during the preceding FB blocks at increasing the bar magnitude.

Following removal from the scanner, participants completed a brief questionnaire. Participants indicated on 5-point Likert scale (Likert, 1932) how much control they felt they had over the bar. The questionnaire also presented a number of strategies (e.g., focusing more on the moving hand, moving faster) and asked the participant to report whether they used those strategies and how effective they felt each strategy was on a 5-point Likert scale.

### Offline data analysis

BOLD fMRI data for each subject were analyzed off-line using tools from the FMRIB software library (http://www.fmrib.ox.ac.uk/fsl). Pre-processing of the images included, motion correction spatial smoothing using a Gaussian kernel of 5-mm full width at half maximum (FWHM), and slice-timing correction.

**Fig. 2:**
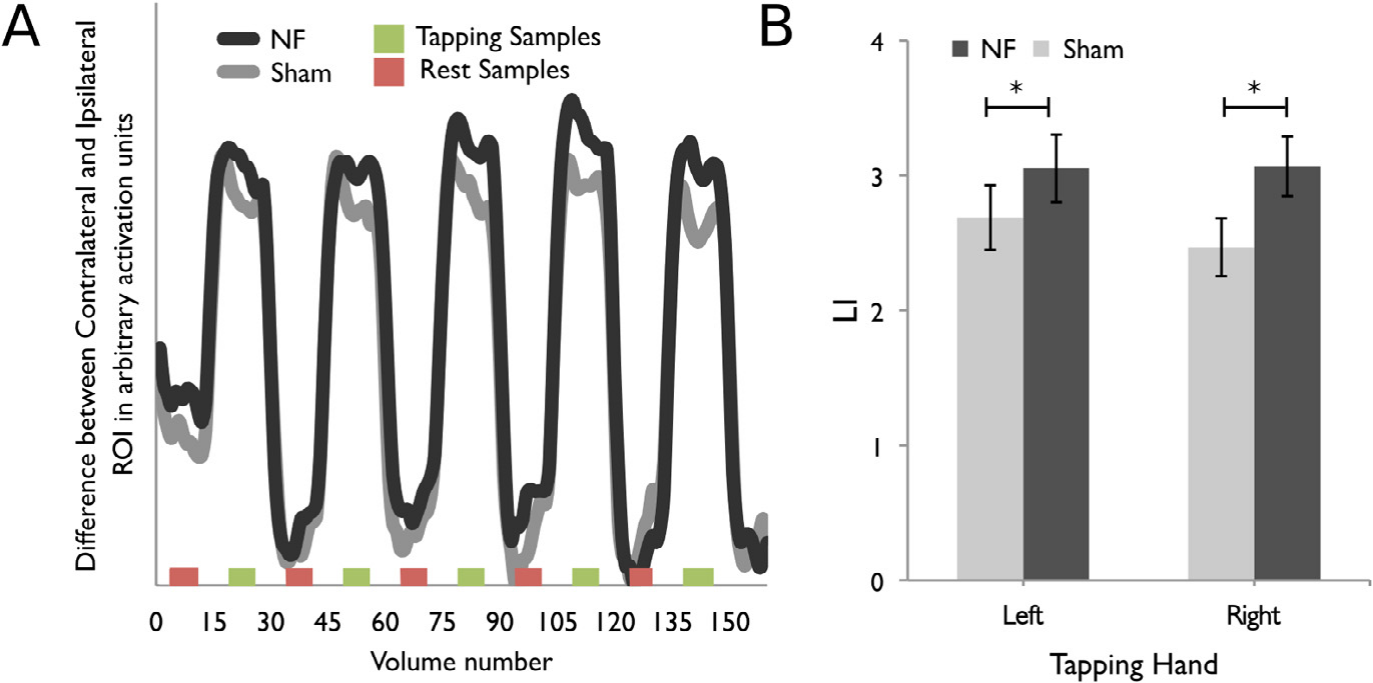
Experiment I. (A) Timeseries of difference between the contralateral and ipsilateral ROI (i.e., LI) for the Sham (gray line) and NF (black line) groups averaged across participants and scans. The red bars along the x-axis indicate the volumes that were sampled from the rest block and the green bars indicate the samples from the tapping blocks that were used to compute PSC in each block (see text for details). (B) LI values averaged over all four FB scans for movement with the left and right hands. There was a significant main effect of group. Error bars are standard error of the mean. ^*^Significant effect of Group.

We calculated an offline LI for each block of each scan. Note that there are slight differences from the calculation of LI for bar width which are detailed in the following paragraph. We first extracted the average time series across voxels within each of the motor ROIs for each participant in both the NF and Sham groups. The PSC associated with tapping in each block was calculated by taking the difference between the average signal over the central eight volumes of the tapping block and the average signal during the central eight samples of all of the rest blocks and scaling by the activity during all of the rest blocks in the scan. (Fig.2A). We started the sample eight volumes after the initial task instruction. The first four volume shift accounted for the hemodynamic delay and the further four volume shift was done to ensure sampling of the central eight volumes of the 15 volume tapping block. The central eight volumes of each block were selected because this was the most stable point in the signal (i.e., the activation was not in the process of changing from rest to active or vice versa). The average ipsilateral and contralateral PSC and LI was calculated for each of the FB scans (or for each hand in the scans in the case of the Pre and Post NF scans). The PSC from ROIs contralateral and ipsilateral to the hand moved was used to calculate an LI (Contralateral PSC – Ipsilateral PSC). Note that this equation focuses on contralateral vs. ipsilateral activation instead of the left vs. right activation used for the NF display. The left vs. right calculation was used for the display to make the feedback bar intuitive for the participant (i.e., moves more to the left during left-handed movements).

For the feedback scans, contralateral and ipsilateral PSC and the magnitude of the LI were submitted to separate two Group (NF, Sham), × 4 Scan mixed ANOVA, with Group as the between subjects factor. For the Pre and Post-NF test, the contralateral and ipsilateral PSC and the magnitude of the LI were submitted to separate two Group (NF, Sham), × 2 Hand (Left, Right) × 2 Scan (Pre, Post-NF) mixed ANOVA, with Group as the between subjects factor.

Mann–Whitney non-parametric tests (Mann & Whitney, 1947) were used for comparisons between the NF and Sham group on the questionnaire scores.

## Experiment II methods

Methods are broadly similar to Experiment 1 with a few key changes. First, we tested older adults, who are more similar in age to typical stroke patients. Second, participants only trained with one hand, allowing for an increased amount of neurofeedback training for that one hand. Further, training with a single limb is more similar to the paradigm that would be used with stroke patients who would only be training with their affected limb. We chose the left hand because Experiment I showed no transfer for left hand movements following NF removal thus it was of interest to determine whether more training could induce transfer in the left hand.

The way sham feedback was implemented was also changed in Experiment II. The use of control brain areas to provide sham feedback may have frustrated participants in the sham group in Experiment I given that they reported feeling as if they had less control over the feedback than participants in the NF group (detailed in following results section). In Experiment II, the Sham feedback consisted of a replay of the feedback from a yoked participant in the NF group. Finally, the study was conducted on a 3T scanner (whereas Experiment I used 7T) as the increased bore size and less stringent safety requirements of the 3T scanner would make it more amenable for NF training in a stroke patient population.

### Participants

Eighteen healthy older adults over 50 years old were recruited from the community (age range: 50–77 years; median age: 67.5 years; 11 males, 1 left handed). All participants provided informed consent and the procedures were approved by the local ethics board (University of Oxford Central University Research Ethics Committee, approval reference: MSD-IDREC-C1-2012-151).

Participants were equally distributed between a neurofeedback (NF) group (age range: 50–75 years; median age 64 years, seven males) or a Sham group (age range: 52–77 years; median age: 69 years, four males). There was no significant difference in age between the NF and Sham groups (*p* > 0.10). Similar to Experiment I, participants were not aware of their group assignment and a blinded experimenter provided all instructions to the participant.

### Procedure and offline data analysis

Unless otherwise specified in the following section, the procedure and analysis were the same as Experiment I. Imaging was performed on a 3.0T Siemens Verio MRI system (Siemens, Erlangen, Germany). All fMRI scans were performed using a 35-slice (3-mm) axial plane, gradient EPI acquisition, with 3 × 3 mm^2^ in plane resolution (TR = 2000 ms; TE = 30 ms; flip angle = 90° FOV = 192 × 192 mm; matrix = 64 × 64). The feedback was projected to the participant in the scanner using a 1920 × 1080 pixel screen with a 60-Hz refresh rate.

To localize the ROIs for feedback, participants were instructed to tap their fingers in sequence from index to little finger during tapping blocks (12 s) which were interspersed with rest blocks (24 s). Participants completed three tapping blocks for each hand, first three blocks for the right hand then three blocks for the left hand (Pre-NF Scan) (rather than alternating between hands for each tapping block as was done in Experiment I). 15 × 15 × 9 mm ROIs were centered over the peak of activation in left and right hand knob. In the NF blocks, participants only used their left hand and completed four feedback scans with the rest and tapping blocks having similar timing to Experiment I. For participants in the Sham Group, a replay of a matched participant’s data was presented to the participants using the custom-made plug in for Turbo-Brain Voyager combined with developed stimulus presentation software which allowed the Sham Feedback to be triggered in a similar manner to the real NF. The Post-NF scan was identical to the Pre-NF scan.

The older adults had increased head motion with hand movement, therefore, prior to the offline PSC and LI calculations, FIX, a FSL based tool to autoclassify noise components was used to correct for noise components including motion in the data (Griffanti et al., 2014, Salimi-Khorshidi et al., 2014). For the feedback scans, contralateral and ipsilateral PSC and the magnitude of the LI were submitted to separate two Group (NF, Sham), × 4 Scan mixed ANOVA, with Group as the between subjects factor. One participant only completed three NF scans thus was removed from the repeated measures analysis of training effects. For the Pre and Post-NF test, the contralateral and ipsilateral PSC and the magnitude of the LI were submitted to separate two Group (NF, Sham), × 2 Hand (Left, Right) × Scan (Pre, Post-NF) mixed ANOVA, with Group as the between subjects factor.

## Experiment I results

### Neurofeedback scans

Consistent with our hypothesis, the young NF group had a larger magnitude LI than participants in the Sham group during the FB scans, *F*(1,23) = 4.37, *p* < 0.05 (Fig.2A, B). No other main effects or interactions involving Group were significant for LI. No main effects or interactions were significant for PSC in the contralateral ROI or ipsilateral ROI during the feedback scans. Thus there is only a difference between the two groups when the two ROIs are considered together as a LI, the metric that was fed back to the participants.

### Pre and post test scans

**Fig. 3:**
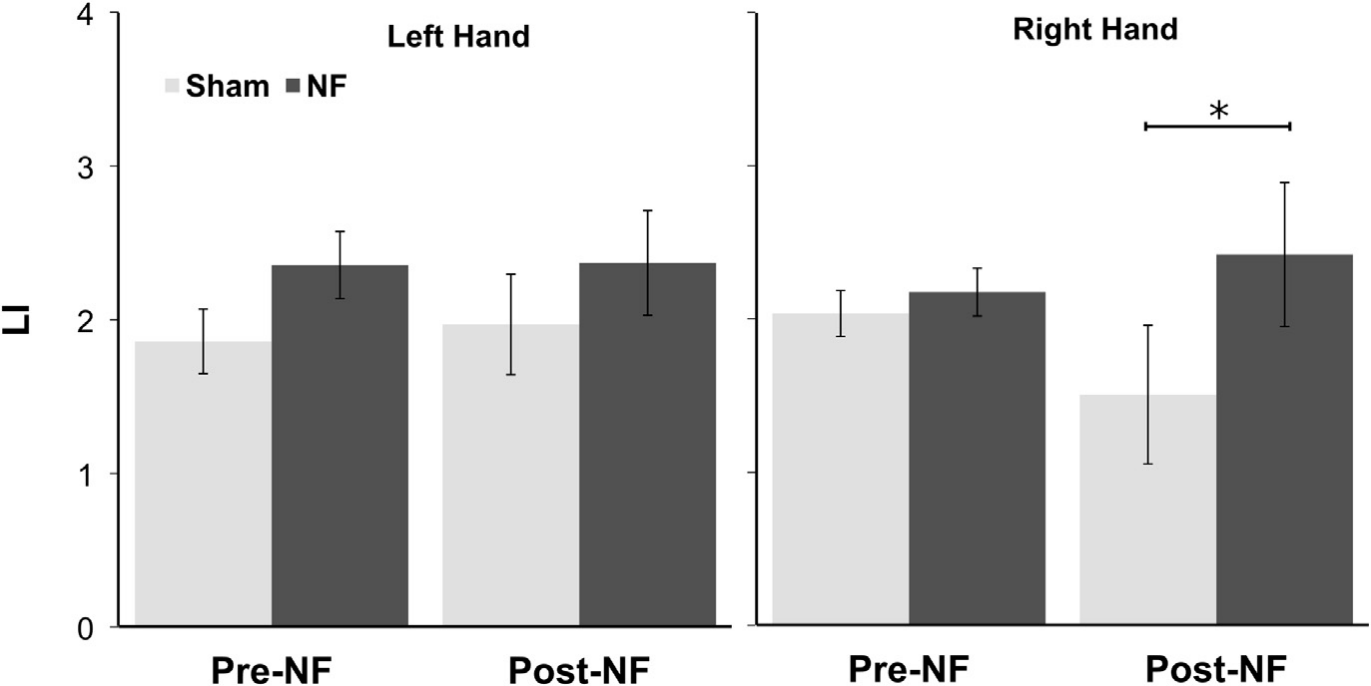
Experiment I. LI values for the Pre and Post-NF scans for the left and right hand, Error bars are standard error of the mean. ^*^Significant effect of Group.

Also important for our hypotheses, in the Pre and Post-FB scans there was a significant interaction between Time, Hand and Group, *F*(1,23) = 4.55, *p* < 0.05 on LI (Fig. 3). The young NF group had a significantly larger magnitude LI during the right hand tapping blocks in the Post-feedback scan (Tukey’s CV = 0.57) indicating that there was some transfer from the feedback training once feedback had been removed for the right hand. For LI, no main effects or other interactions were significant.

For PSC, we did not find clear evidence for effects of feedback. In the contralateral ROI, no effects involving Group were found. In the ipsilateral ROI, a three-way interaction between Time, Hand and Group, *F*(1,23) = 4.68, *p* < 0.05, was found, but this was driven by significantly higher ipsilateral activation for the left hand in the Sham group during the Pre-feedback scan (Tukey’s CV = 0.49). No other main effects or interactions were significant.

### Questionnaire

Participants in the Feedback group felt they had more control over the feedback than participants in the Sham group, *U*(24) = 27.0, *Z* = 2.92, *p* < 0.05, (NF Mean: 3.41, Range: 1–4; Sham Mean: 2.15, Range: 1–4). Further, more participants in the Feedback group reported having greater control over one hand than the other (9/12 participants) than participants in the Sham group (5/13 participants), a difference that approached significance, Z = 1.98, two-tailed *p* < 0.07). The majority of participants reported they felt more in control of the feedback when using their dominant (right) hand. With regards to participants’ rankings on how useful the strategies they tried were, there was no difference in the rankings between the questions for participants in the NF group (*ps* > 0.05).

## Experiment II results

### Neurofeedback scans

**Fig. 4:**
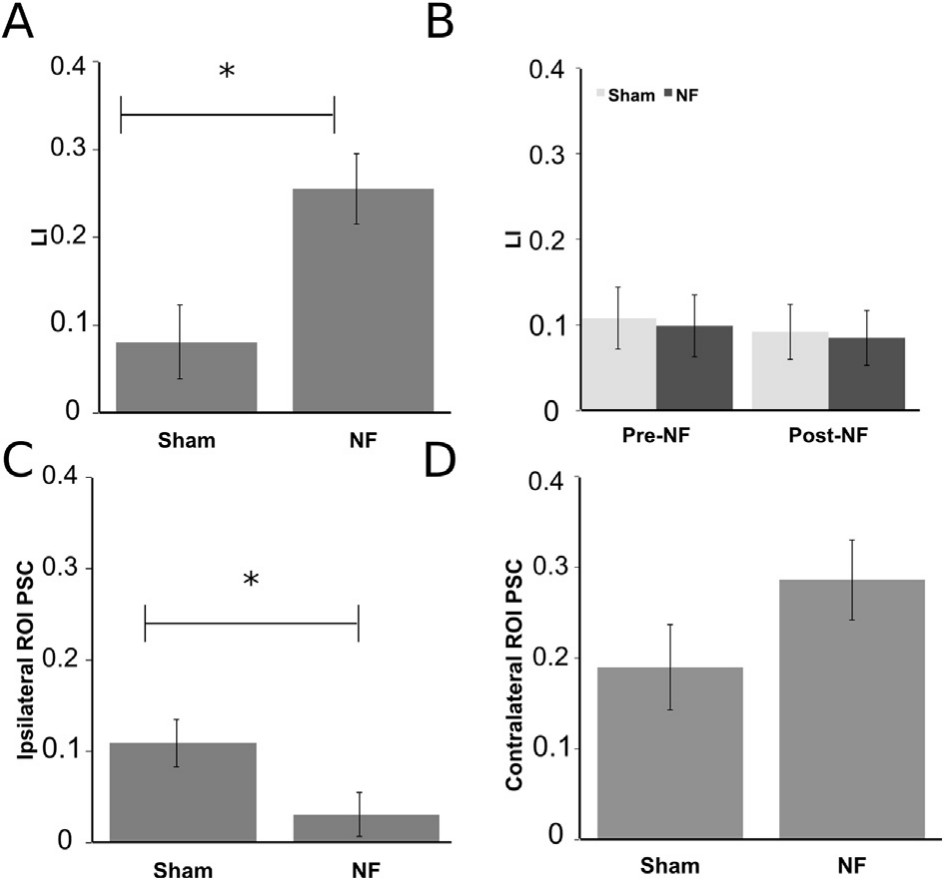
Experiment II. (A) Significant difference in laterality index (LI) between the Sham and NF groups during NF training. (B) LI for each group during the Pre/Post-NF scans. (C) Significantly larger ipsilateral activation in the Sham group during NF training. (D) Contralateral activation in Sham compare to NF group. All error bars are standard error of the mean. Note that PSC and LI values are lower in Experiment II compared to Experiment I because a 3T (instead of 7T) scanner was used and FIX (see text for details) was used to auto-classify and remove noise components from the data.

Consistent with our hypotheses, the older NF group had a significantly larger magnitude LI than the Sham group during neurofeedback scans, *F*(1,15) = 9.08, *p* < 0.01, (Fig.4A). No other main effects or interactions were significant for LI.

No significant main effects or interactions were found for the neurofeedback scans for the PSC in the contralateral ROI. In the ipsilateral ROI, the NF group had a significantly smaller PSC than the Sham group, *F*(1,15) = 4.77, *p* < 0.05, (Fig.4B) indicating the difference in the LI may have been driven by lower activation in the ipsilateral hemisphere. No other main effects or interactions were significant for PSC in the ipsilateral ROI.

### Pre- and post-test scans

Our primary interest in comparing Pre- and Post-NF scans was to test if the increased laterality achieved during NF training persisted after the removal of feedback. We would predict this to be evident by either a Group (NF, Sham) by Scan (Pre, Post) interaction where the NF group would have a larger LI following training in the Post-NF scan or a Group (NF, Sham) by Scan (Pre, Post) by Hand (Left, Right) interaction where an improvement would only be seen in the trained, left hand. The Group by Scan interaction was not significant *F*(1,16) = 0.002, *p* > 0.10 for LI suggesting that the neurofeedback effect seen during training did not persist to the Post-NF scan (Fig.4C) nor was the three-way interaction *F*(1,16) = 0.62, *p* > 0.10.

There were no significant main effects or interactions for LI or the ipsilateral ROI PSC. There was a significant interaction between Group and Hand for the PSC in the contralateral ROI, *F*(1,16) = 5.80, *p* < 0.05. Post hoc testing revealed greater activation for the left (M = 0.31, SEM = 0.10) compared to right hand (M = 0.18, SEM = 0.10) in the NF group, *t*(8) = 4.34, *p* < 0.01 but no significant difference between hands for the Sham group, *t*(8) = 0.20, *p* > 0.10 (left, M = 0.40, SEM = 0.10; right, M = 0.41, SEM = 0.10). Reasons for this particular interaction are unclear but given that the absence of any interaction with scan, and the lack of any baseline differences in activity between groups, we do not consider the group × hand interaction relevant to the neurofeedback effect or its generalization.

### Questionnaires

There was no significant difference between the NF and Sham group in how much control they felt they had over the feedback bar (NF Mean: 2.72, Range: 2–4; Sham Mean: 2.5, Range: 1–3.5) or between the effectiveness of any of the strategies queried on the questionnaire (all *ps* > 0.10).

## General discussion

Taken together, we have shown in two different samples, that participants can use a NF signal to control the laterality of their brain activity while executing movements. Further, this effect was present across two different age ranges, and importantly was present in a cohort of participants that had a similar age range as many people who have had a stroke. With regards to potential clinical application, it was less encouraging that the NF effect did not consistently persist following removal of the NF. Each of these results will be discussed in turn.

### Neurofeedback training

In both experiments, participants who received real NF had more lateralized motor activity during NF scans than those in the Sham group. The finding that participants can modulate the activity in motor cortex using neurofeedback is consistent with the findings of a number of previous studies. However, the majority of previous studies have used motor imagery tasks (e.g., Auer et al., 2015, Chiew et al., 2012, deCharms et al., 2004, Yoo et al., 2008).

By contrast, the present study required participants to physically perform the movements rather than engage in motor imagery. The use of physical movements may have made it more difficult to find a difference between the two groups as the participants in the Sham group would be expected to and did have strongly lateralized activity in motor ROIs while moving their hand compared to rest. Thus participants in the NF group were able to further alter the activity in the motor ROIs beyond the Sham group’s already high level of activation, even though both groups had been given the same instructions for movement strategies by a blinded experimenter. In contrast, motor imagery is a more difficult task to perform, and many studies have suggested that this task does not typically engage the primary motor cortex (e.g., Hanakawa et al., 2005). Thus a proportion of participants are not able to increase activation in the motor regions of the brain significantly above rest levels during motor imagery, with or without feedback (e.g., Auer et al., 2015, Berman et al., 2012, Chiew et al., 2012; c.f., Bray et al., 2007). The task used in the present study therefore allowed for a strong test of participants’ ability to use neurofeedback as all participants were able to perform the task and activate motor cortex, but with the participants in the NF group showing better performance at manipulating the LI than those in the Sham group.

Note that there was no effect of scan of any of the brain activity measures in the current study, indicating that NF group was quickly able to use the feedback signal to alter their brain activity and that no further improvements were seen across scans. This lack of any apparent learning over scans may have been due to ceiling effect, or the task may not have been challenging enough. For instance, the percent signal change associated with the maximum bar width could have been increased at a greater rate to make it more difficult for participants to increase the bar width. Finally, further improvement may have been seen with more training such as increasing the number of session across days, an issue that will be discussed more in the following section regarding transfer to the Post-NF scans.

Our results show that both younger and older adults were able to increase LI through neurofeedback. LI contrasts activity across the two hemispheres so we aimed to unpack this effect by investigating signal change within the ipsilateral or contralateral ROIs. For the younger adults in Experiment I no significant effects were found, whereas for the older adults in Experiment II, there was reduced activation in the ipsilateral ROI in the NF group compared to the Sham Group. Of note, Berman et al. (2012) found no difference between a NF and no NF group during motor execution when the feedback derived only from the signal in the contralateral hemisphere. Moving forward, displaying activity in the contra and ipsilateral hemisphere separately may encourage strategies that both increase activity in the contralateral hemisphere and decrease activity in the ipsilateral hemisphere, leading to even greater lateralization. A possible reason for reduction in ipsilateral activity being particularly evident in the older sample is that older adults tend to have a more bilateral pattern of activation compared to younger adults with increased activation in the ipsilateral motor regions (Ward and Frackowiak, 2003).

Maintaining a difference in activation following NF removal is a key requirement in a therapeutic context. We found mixed evidence for such transfer, with evidence for transfer for the right hand only for the younger adults. Older adults only trained with their left hand and similar to the younger adults, saw no transfer of altered brain activity following NF removal. All but one of the participants were right handed, thus it may be that transfer occurs more readily for the dominant hand; however, we are unable to conclude this in the older adult population as NF training with the right hand was not undertaken for this group. As we will elaborate on further below, the use of a 3T vs. a 7T scanner for the older adults is a limitation to comparison between the two studies. The use of the 3T scanner may have reduced our ability to detect a transfer effect in the older adults compared to the younger adults.

Regardless of hand dominance, more neurofeedback training may be necessary for participants to maintain the altered activation patterns. To minimize fatigue this could be delivered over multiple sessions rather than longer sessions. For instance, Auer et al., 2015 found that participants were able to maintain increased lateralization following NF removal after 12 sessions spread over four weeks. Another possibility is for patients to practice the task outside of the scanner following an initial NF session. For example, Yoo et al. (2008), provided young adults with neurofeedback from contralateral motor cortex while performing motor imagery. Participants then practiced the motor imagery task daily at home for two weeks and participants were able to maintain the level of activation seen in the presence of neurofeedback after the neurofeedback was removed.

Another factor that may have limited our ability to detect transfer effects was differences in the timing and movement conditions between the Pre/Post-NF scans compared to the NF scans. For instance, in Experiment I, a blocked practice schedule was used where each scan consisted of participants only using one hand. The Post-test however had a mixed test schedule with participants alternating between using their right and left hand on each tapping block within the scan. Previous learning research has demonstrated that the best performance in transfer test results from mixed-schedule practice with a blocked-schedule transfer test whereas the worst performance in transfer tests results from blocked-schedule practice and mixed-schedule transfer (e.g., Brady, 2004, Magill and Hall, 1990). In addition, in both experiments, the tapping–rest block cycle in the Post-NF scan was shorter (12 s tapping/ 24 s rest) compared to that used in training (30 s tapping/ 30 s rest). The difference in timing between the NF scans and the Pre and Post NF scans is a limitation in the design because it restricts to comparisons and conclusions that can be drawn from comparing between NF training and after NF removal.

Similarly, we are limited in direct, statistical, comparisons of the laterality index and PSC values between the younger and older adults because different strength scanners (7T for the younger adults and 3T for the older adults) were used between the two Experiments and therefore different levels of signal change, and therefore LI, would be expected (e.g. LI values in Figs. 2B and  4A differ substantially). Previous work comparing EPI signal in the motor cortex has shown that and increase field strength increases signal-to-noise ratio (SNR) (e.g., van der Zwaag et al., 2009). An increase in SNR with increased field strength could provide improved rt-FMRI NF; however, the decreased bore size and more stringent exclusion criterion at higher field strengths may not be amenable for a stroke patient population. Thus it was encouraging that our older adult population was able to use the NF from the 3T scanner.

Similar to other studies (e.g., Bray et al., 2007, Chiew et al., 2012, deCharms et al., 2004, Yoo et al., 2008) the present study used sham control groups. Compared to a ‘no feedback’ control group, sham feedback allowed the experimenter to recreate a similar visual environment and to provide the same instructions and possible modulation strategies. In the first experiment the use of control brain areas to provide sham feedback may have frustrated participants in the Sham group. Consistent with this, participants in the sham group for Experiment 1 reported lower feelings of control over the feedback signal than the NF group. The second experiment used a different control condition, presenting NF from a yoked participant as Sham. In this case, participants in the Sham group were seeing the same stimuli (and receiving the same impression of improving performance) as the NF group, but the changes in the NF display did not match the changes in their brain activity (and their brain LI did not increase). In Experiment 2, both the NF and the Sham groups reported a similar level of control of the NF signal thus yoked feedback may better match feelings of feedback control between the two groups. As noted previously, in both experiments the NF had more lateralized activity than the Sham group thus the type of Sham may play a minimal role in the determining the effects of neurofeedback on LI. This observation is consistent with the finding from a study that used RT-fMRI neurofeedback to down-regulate the rostral anterior cingulate cortex to reduce pain (deCharms et al., 2005). A feedback group was compared to four different control groups included a no-feedback group, a yoked-sham group where the feedback was based on another participant’s feedback and feedback from an unrelated brain area. The feedback group had better performance than all of the control groups, which performed similarly.

## Conclusions

The current study demonstrated, in two different samples, that healthy adults can use fMRI neurofeedback to increase laterality of motor activity during movement execution. In particular, given that our sample in the second study was a similar age to stroke patients, the result provides evidence that neurofeedback may be an effective mechanism for rehabilitation following stroke. Further research is required to evaluate the fMRI NF paradigm in patients with stroke to enhance the effectiveness of physical practice in those who retain at least some limb mobility. To allow for application to a broad range of contexts, future research could also explore the possibility of using alternative sensory modalities (e.g., auditory, haptic) and imaging modalities (e.g. electroencephalography) for feedback (See Sitaram et al., 2016 for review). Evidence of improved long-term functional outcomes will be key to demonstrating success of this approach and facilitating widespread adoption.
